# What stands behind the gender gap in entrepreneurship? Untangling the intergenerational parental role

**DOI:** 10.1371/journal.pone.0261108

**Published:** 2021-12-21

**Authors:** Yaron Zelekha

**Affiliations:** Faculty of Business Administration, Ono Academic College, Kiryat Ono, Israel; Politecnico di Torino, ITALY

## Abstract

This research examines the entrepreneurship gender gap by offering an additional novel explanation for the higher share of men in entrepreneurial activity focusing on intergenerational parental role. Participants (N = 1288) aged 18–81, including 259 actual entrepreneurs, completed questionnaires about entrepreneurship tendency, personality traits and socioeconomic background. The gender gap in actual entrepreneurship continues a significant difference in entrepreneurial tendency, which is developed in the first and the second stages of the entrepreneurial trajectory. When women reach the third stage of entrepreneurial development, the execution stage, they have already acquired a self-perception of an incapable and incommensurate entrepreneurial personality. The results indicate that role modeling behavioral channel significantly accounts for the gender gap in entrepreneurial personality. The results suggest that both parents contribute to women’s’ inferior perception of entrepreneurial personality and that their contribution affects all four aspects of the entrepreneurial tendency. It appears that the impact of fathers’ role modeling is larger than that of mothers, and furthermore fathers transfer other entrepreneurial role models from their side in the family.

## 1. Introduction

Women face discrimination in labor markets. The gender discrimination is partly explained by several factors, among them: wage discrimination; smaller participation of women in high wages markets; promotion discrimination; difference in working hours since women take a larger share of family tasks; and even a form of discrimination exercised by women themselves, who, often unknowingly or due to fears of negative responses, ask for lower wages then men for the same positions [1].

Under this framework, entrepreneurship is often promoted by many countries as an important source of alternative employment for women [2]. However, women take smaller part in actual entrepreneurship for reasons which are only partially explained [3, 4]. Moreover, the literature point that the gender gap in actual entrepreneurship does not decrease even in labor markets characterized by high gender equality and tends to be robust across cultures and national boundaries [5].

Two explanations for the gender gap in actual entrepreneurship are suggested:

First, the limited entrepreneurial activity among women may reflect low tendency to become an entrepreneur. If that explanation is right, it is expected that similar gender gap will exist also at all the three stages of the entrepreneurial development. However, this assumption has not been examined yet. In this regard, the entrepreneurship literature describes three major stages of entrepreneurial development:

1. Discovery or opportunity identification [6–9];
2. Opportunity (problem-solution) validation and development [10–12];
3. Execution of actual entrepreneurship by allocating resources [6, 8, 13].

Second, the limited entrepreneurial activity among women may reflect discriminatory practices and social exclusion that take place only at the execution stage. According to these accounts, women who want to participate in entrepreneurial activity, are not given equal opportunity to do so, due for example to limited allocation of resources in general and limited access to cash in particular (for the importance of access to cash see [14, 15]). Therefore, it can be inferred that woman tend to participate in entrepreneurial activity in the first two stages of the entrepreneurial development, but their entrepreneurial development is impeded at the execution stage.

This paper will use a novel empirical approach in order to uniquely explore in which entrepreneurial developmental stage starts the entrepreneurial gender gap and what are the parental roles for this gap.

In the first study, I use very large data sets of 1288 participants. Among them 321 triads (963 participants) comprise of an adult with both of their parents. All assessed for their actual entrepreneurship activities as well as their entrepreneurial tendency. While the former measures the third stage of entrepreneurial development, actual entrepreneurship, the latter focuses on the first and second stages of entrepreneurship development.

The purpose of this study is to examine whether the entrepreneurial gender gap reflect societal barriers for women to engage in the third stage of entrepreneurial development, actual entrepreneurship, or does it reflect a deeper problem originates at the first and second stages of entrepreneurship development.

If the entrepreneurial gender gap is the outcome of discriminatory practices and social exclusion that begin only at the execution stage, then the gender gap will be significantly smaller at the entrepreneurial tendency stages that precede the actual entrepreneurship stage. On the other hand, if the entrepreneurial gender gap is as large in the entrepreneurial tendency as in the actual entrepreneurship, then it may emerge very early at life.

The second study focuses on parental influence, which is suggested as a crucial factor explaining the gender gap. I use portion of the data from Study 1, a set of 321 321 triads, in order to control for and examine the possible parental effects on the entrepreneurial gender gap above and beyond the effects of individual personality, socio demographic and cultural characteristics.

Parental practices affect significantly actual entrepreneurship, such that having a parent who is an entrepreneur increases the probability that the descendant will be an entrepreneur by 30 to 200 percent [16–18].

The explanations to these findings [19, 20] were divided between the transmission of familial resources including human capital and the transmission of preferences, attitudes, and parental role modeling. However, the former explanation was hardly supported by the empirical evidence and definitely not as primary factors [16, 18, 20, 21]. While, very limited studies dealt with the latter explanation.

In this regard, several studies establish the importance of role model on entrepreneurship. Parental role model [22] and mentor role models [23, 24] effects were found regarding entrepreneurship for both biological and adoptive parents and especially of the nurturing parent of the same sex [18]. Indications were also found for the presence and importance of entrepreneurial role models on actual entrepreneurs, which were dependent on the strength of the relationship between the entrepreneur and his/her role model [25]. Furthermore, support was found for an intergenerational transmission of the value of Mastery among entrepreneurs [21].

The major shortcoming of the papers examining parental role modeling is that they did not differentiate between three possible parental effects:

1. General environment such as general cultural values and attitudes that may promotes entrepreneurship that are mediated also by the parents (the cultural channel) [26].
2. Inherited personality traits as well as inherited entrepreneurial tendency [27] that may affect entrepreneurship. Indeed, [28] claim for a heritability of 40–50% of the Big-Five personality traits (the personality channel).
3. Parental behavioral effects since individuals tend to learn by observing the actions of their parents and transforming these cues into internal codes, which form part of the offspring’s’ mental models and can lead individuals to initiate similar behavior (the behavioral role model channel) [29, 30].

The second study aims to address this gap by controlling both heterogenetic cultural environments as well as the parents’ personality, to isolate the parental behavioral entrepreneurial role modeling. A family design is suitable for entangling these three possible parental effects since the data set will allow to measure parental entrepreneurship personality, parental entrepreneurial cultural and entrepreneurial role modeling.

This research makes three important contributions to the research on gender differences in entrepreneurship. First, it offers a novel empirical approach, which will allow a better understanding of the role of parental, social, economic, culture and personality drivers for gender differences. Second, I add to the empirical literature on entrepreneurial gender gap by examining both actual entrepreneurship as well as entrepreneurial tendency for the same data set. Third, I suggest part of the unresolved gender gap in entrepreneurship is an outcome of deeper drivers than just the discriminatory practices typify the execution level.

## 2. Related literature and general hypotheses

### 2.1 Gender and entrepreneurship

As reviewed above, women are less involved in entrepreneurship than men. Few studies tried to test whether there are any psychological differences between women and men entrepreneurs and whether these differences can explain the entrepreneurship gender gap. In this regard, men reported higher opportunity evaluation than women when gender stereotypical information was presented and affected their performance, whereas man and women evaluated business opportunity equally favorable while no gender stereotypical information was presented [31, 32].

Indeed, although men and women use fundamentally different processes of opportunity identification, there is no difference in the level of innovativeness of the opportunities identified [33].

Furthermore, women entrepreneurs are more likely than men to emphasize social value goals over economic value creation goals [3]. These findings may suggest that women have entrepreneurship abilities, but they divert them into environment that are more related to social targets such as intrapreneurship in the public sector or in NGO’s rather than business entrepreneurship.

The literature described may suggest two different theoretical claims.

On the one hand, since even modern and western societies are characterized to significant extent by gender stereotypes, women cannot be detached from gender stereotypes outside the laboratory. Therefore, it hard to believe that growing up in gender-stereotypical environment will not affect women in the development of entrepreneurial personality.

It is possible that women internalize gender stereotypes in which entrepreneurship is a manly character, and as a result it is expected that their self-perception excludes being an entrepreneur. Indeed, women tend to perceive themselves as well as their environment as less favorable for entrepreneurship compares to men across numerus countries [2]. Assuming that internalization of stereotypical perceptions that deter women from entrepreneurship start at early age, it is suggested that gender differences exist at the first two stages of entrepreneurial development.

On the other hand, it is also possible that women have similar entrepreneurship level as men do in the first two stages of the entrepreneurial development, but they only partly proceed into the third level of actual business value creation of entrepreneurship. This is because the discriminating environment against women characterizes the execution stage of entrepreneurship. For example, discrimination in credit allocation or in the ability to recruit investors, partners and even employees or customers. Therefore, the momentum started at the first two stages of entrepreneurial development is shifted to other areas of entrepreneurship, such as public sector intrapreneurship (that is entrepreneurship inside an already existing unit of operation).

Therefore, it is highly important to understand whether the entrepreneurial gender gap is fully presented in all three stages of entrepreneurial development. It is reiterated that the literature has focused mostly on the execution stage and thus is not being able to fully address the entrepreneurial gender gap. However, the limited literature may suggest that women internalized to certain extent entrepreneurial gender norms, which for some women divert their attentions away from business entrepreneurship.

Thus, I hypothesized that:

*H1*: *Women gender will have negatively significant associations (or alternatively Men gender will have a positively significant associations) with both actual entrepreneurship (a measure of the third stage of entrepreneurial development) and the tendency to become an entrepreneur (a measure of the first two stages of entrepreneurial development)*. *Thus*, *expressing the entrepreneurial gender gap in all three stages of entrepreneurial development*.

In addition, employment related variables (being a part time worker) and to a lesser degree family related variables (marriage, number of children, their gender, and their ages) contributed to the gender differences in entrepreneurship [34].

Thus, I hypothesized that:

*H2*: *Social variables (such as having a family*, *older age and being religious) will moderate the association between gender (women) and entrepreneurship such that they increase the expected negative association*.
*H3*: *Economic variables (such as being a salary employed and low income) will moderate the negative association between gender (women) and entrepreneurship such that they increase the expected negative association)*.

In this regard, I will also examine the possible association between education and entrepreneurship, although prior research did not find any clear such associations [2, 35]. This is because education can used as a proxy for income.

### 2.2 Culture and entrepreneurship

The research on the effects of social and cultural forces on entrepreneurship can be traced back to very early papers on entrepreneurship since the pioneering claim for the Protestant work ethic [36]. Modern papers suggested that specific country variables such as economic freedom or culture of social spending seem to encourage entrepreneurship [37]. Other claimed that religions have built-in mechanisms for the perpetuation of values, regardless of whether a person is religious Indeed, empirical evidence were found that a country’s entrepreneurship level is determined mainly by its major religion of the country and that the relative sizes of the different religious groups have no effect [26]. Thus, suggesting that the macro effects of religion on the countries level of entrepreneurship are more likely associated with the general public cultural channel, which is shared by both the majority and the other minority religion’s members. Needless to say, parents are the major cultural agents.

However, the literature on the cultural factors that affect entrepreneurship, to the best of my knowledge, has not examined the role of parents as transmitters of entrepreneurial culture in developing the tendency to become an entrepreneur. Given that the current sample holds constant the Israeli entrepreneurial culture of the children, I can examine the effect of entrepreneurial culture that the parents imported from out of Israel on the children’s’ entrepreneurship. Thus, I hypothesized that:

*H4*: *The cultural legacies of both parents regarding entrepreneurship as measured by the level of entrepreneurship in the parents’ country of birth will have positive significant associations with their child’s tendency to become an entrepreneur as well as on actual entrepreneurship*.

### 2.3 Personality and entrepreneurship

Early research on entrepreneurship looked at entrepreneurs as a uniform class and focused on personality characteristics of entrepreneurs. Since the early 2000’s, a new stream of research stresses the relationship between individual differences in entrepreneurship in four main domains: entrepreneurial personality traits [38–41], attachment orientations [42], entrepreneurial motivation [43] and cognition [44, 45].

Several recent meta-analyses have shown that entrepreneurs do differ from other groups (e.g., managers) in terms of their personalities. For example, [40, 41] conducted a set of meta-analyses to examine the relationship between personality (based on the Big Five model) and entrepreneurial intentions and performance. Results indicate significant differences between entrepreneurs and managers on four personality dimensions: entrepreneurs scored higher on Conscientiousness and Openness to Experience and lower on Neuroticism and Agreeableness. No difference was found for Extraversion. However, the authors focused on the contrast between entrepreneurs and managers, without comparisons to the general population.

However, the literature connecting the Big Five personality and entrepreneurship ignored the role of parents and did not interlink with the literature on intergenerational parental effects. In this regard, the research on attachment orientations, which are primarily driven from parental behaviors, shows clear evidence that attachment orientations not only predict an adult’s tendency to become an entrepreneur, but also which type of entrepreneur he/she will become [42].

In addition, psychological factors may have important role in entrepreneurial process. For example, intentions [38, 40, 41], opportunity recognition [7], motivation and cognition [43–46], and business performance and success [47] all play significant role in developing entrepreneurship. While these psychological constructs can be partly genetically inherited, they are also significantly socially affected starting from early childhood, therefore, stressing the important role of parents as transmission channels.

Regarding the influence of psychological individual differences on gender differences, evidence was found that job autonomy and job security were positively significant predictors for entrepreneurship of women, whereas need for achievement and risk-taking tendency were positively significant predictors for entrepreneurship of men [4]. Other papers highlight gender differences in competitiveness which may serve as a predictor for entrepreneurship since women seems to be less competitively inclined than men [48, 49] and characterized with relatively lower risk taking [50]. However, others found that higher risk aversion of women has only a tiny effect on self-employment [51].

Thus, I hypothesized that:

*H5a*: *Openness to Experience*, *Conscientiousness*, *and Extraversion will have positively significant associations with Actual entrepreneurship as well as with the tendency to become an entrepreneur while Neuroticism and Agreeableness will have negatively significant associations*.
*H5b*: *The positively significant associations of Openness to Experience and Extraversion with the tendency to become an entrepreneur will be moderated by gender*, *such that for women these associations will be smaller than for men*.

It is possible to examine the associations of parents’ personality traits on the entrepreneurial tendency or on the actual entrepreneurship of the child. However, the parents’ personality traits are significantly associated with their own entrepreneurial tendency and therefore it is better to examine directly the associations of the parents’ entrepreneurial tendency and not the parents’ personality traits (see next section).

### 2.4 Parenting and entrepreneurship

In addition to the possible role model effects described earlier, the literature may suggest that general personal characteristics of entrepreneurs might be influenced by parents’ creativity, risk bearing, tolerance for ambiguity and social skills. In this section, I will describe how these characteristics of the parents may be related to entrepreneurship of the child beyond the role modeling effects.

Creativity and entrepreneurship are positively correlated [52, 53]. Individuals come into the world equipped with interrelated behavioral systems. An example is exploration, the urge to go out into the world to work, play, discover and create. However, to develop curiosity and creativity, the infant need to have a secure environment, which is afforded if it attached to its parents in a secure rather than insecure attachment [42, 54]. Indeed, secure attachment orientation enhances curiosity, encourages exploration of new information and phenomena, favors the formation of new and flexible cognitive structures and allows risk bearing and tolerance for ambiguity. This in turn elicits entrepreneurship behaviors [42].

Under this framework, it is possible that parents have an additional effect on their children besides possible role modeling through the effect of their own entrepreneurial personality and its relation to curiosity, exploration of new information and phenomena, the formation of new and flexible cognitive structures and tendency to risk bearing and tolerance for ambiguity (even if their entrepreneurial tendency was not exercised into actual entrepreneurship and therefore cannot be served as entrepreneurial role model). Thus, I hypothesize that:

*H6*: *Being an entrepreneurial parent and especially entrepreneur father (role model effect) will have positively significant associations with their child’s tendency to become an entrepreneur as well as with his or her actual entrepreneurship*.
*H7*: *The tendency of both parents to become an entrepreneur and especially of fathers will have positively significant associations with their child’s tendency to become an entrepreneur as well as with his or her actual entrepreneurship*.

Since, the entrepreneurial gender gap exists also at the parents’ generation it is also possible that the effect of the parents will be larger for sons than for daughters, reflecting parents preserving gender stereotype [55]. Thus, I hypothesized that:

*H8*: *The associations of the parents’ tendency to become an entrepreneur and/or their entrepreneurial experience with their child’s tendency to become an entrepreneur will be significantly larger for their sons then for their daughters*.

The effect of parent role modeling is moderated by parents-children gender match. Fathers affect sons and mothers affect daughters to a higher extent than the effect of fathers on daughters and the influence of mothers on sons [56, 57]. Furthermore, according to the Social Role Theory, social beliefs regarding gender roles are adopted by imitating the parents [58, 59]. In addition, it has been found that sons of self-employed fathers more often enter self-employment than sons of self-employed mothers [16]. Moreover, paternal, and maternal role models’ influence is contingent on the child’s personality trait (i.e., the model’s impact is stronger the less the child is Open to Experience) [30].

Out of family role modeling may have different effect on entrepreneurship than parental role modeling, but they may have also some resemblance. In this regard, there are evidence for gendered peer effects of out of family role models. While men are more influenced by other men, women are more influenced by other women [5]. Other findings indicate that entrepreneurs and their out of family role models tend to resemble each other in terms of the characteristics that facilitate role identification including gender [25].

Therefore, different effects of either parent and the additional effect of gender match (and mismatch) on the tendency to become an entrepreneur can be expected. Thus, I hypothesized that:

*H9*: *The gender match (mismatch) of the parent–child*, *will have a significant positive (negative) association between the parent tendency to become an entrepreneur and/or their entrepreneurial experience and the child’s tendency to become an entrepreneur*.

**Fig 1:**
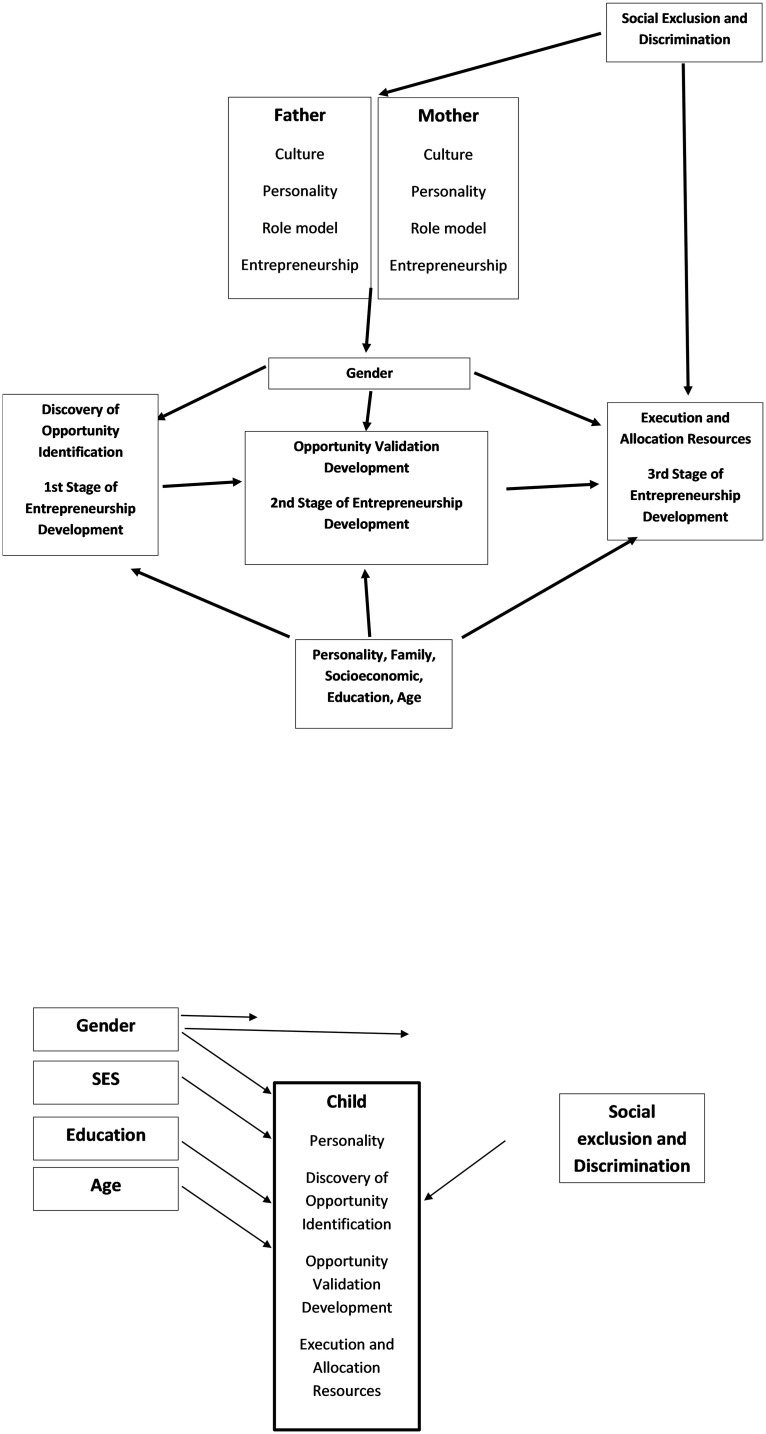
Intergenerational theoretical model.

Fig 1 presents the theoretical model that will be empirically analyzed.

## 3. Data and methods

The following studies were approved by Ono College Ethics Committee approval ono201902. All procedures performed in the studies involving human participants were in accordance with the ethical standards of the institutional and/or national research committee and with the 1964 Helsinki declaration and its later amendments or comparable ethical standards. This article does not contain any studies with animals performed by the author. Informed consent was obtained from all individual participants included in the studies.

### 3.1 Participants

1288 Hebrew-speaking adults (718 women, mean age = 42.1, SD = 16.07, aged 18–81) took part in the two studies. Among them 321 triads of a student for B.A. or M.A. with data for both of his/her parents (963 participants) and additional 325 students without data of their parents. Out of the total 1288 participants, 259 were part time or full-time entrepreneurs, and 1029 were non-entrepreneurs. The data was collected at Ono Academic College, which is the largest private academic institution in Israel, between 2016 and 2019 and were tested for other hypotheses elsewhere. Students received a bonus of 5 points in one of their courses if they and their parents completed the survey. If one of the parents refused to complete the survey the bonus was not provided. A research assistant contacted the parents directly in order to send them the survey and collect it after completion. The response rate of the parents was very high (over 95%), ruling out a potential bias due to different response rate between students and their parents.

I used the nQuery software (https://www.statsols.com/nquery) to determine the strength of using the sample and possible sub-samples. The minimum size of a sample/sub sample was calculated based on the mean and standard deviation of the entrepreneurial tendency score. For a 9-points interval out of a maximum score of 325 points (a third of a standard deviation) a sample/sub-sample should include at least 78 participants, for a 6-points interval the sample/sub-sample should include at least 172 participants, and for a 4-points interval, the sample/sub-sample should include at least 384 participants (two-sided interval test, 95% confidence level).

Table 1 shows the demographic characteristics of the sample while a minimal data set is included in the S1 File.

**Table 1:** Summary statistics.

Variable	Average/Share	SD	Median
Women/Total	717/55.7%	-	-
Age	42	16.07	44
Religious	576/44.7%	-	-
Jewish religion	1200/93.2%	-	-
Born in Israel	965/74.9%	-	-
Having children	866/67.2%	-	-
Years of education*	13.66	3.24	14
Net household income**	13,241	10,195	8,500
Having a father entrepreneur	341/26.5%	-	-
Having a mother entrepreneur	124/9.6%	-	-
Employee	897/69.6%	-	-
Unemployed	88/6.8%	-	-
Entrepreneur	259/20.1%	-	-
Home maker	53/4.1%	-	-
Took a course on entrepreneurship	141/10.9%	-	-

* Very close to General Israeli population, which has a median of 13 in 2018.

**Compared to 15,427 NIS in the General Israeli population for the year 2016. The difference is due to over-representation of young adults with very low income. When correcting for this over-representation, the average net household income was very close to the average general Israeli population.

### 3.2 Materials

#### 3.2.1 Entrepreneurship tendency scale [60]

This self-report scale consists of 65 items that assess four aspects of entrepreneurial personality: Entrepreneurial Awareness/Proactivity (e.g., "I am quick to spot ways of making money"), Entrepreneurial Creativity (e.g., "Even if I know how to do something, I would always try to do it in a different way"), Opportunism/Motivation (e.g., "When I see a business opportunity I jump on it without giving it much thought"), and Vision (e.g., "I am destined to make a difference in the world"). Respondents were instructed to rate each item on a 5-point Likert scale that ranged from completely disagree (1) to completely agree (5). Cronbach’s α for the total scores was 0.90.

The entrepreneurship tendency scale measures the self-perception of participants regarding the first two stages of entrepreneurial development (Discovery or opportunity identification and Opportunity (problem-solution) validation and development) by examining four aspects of entrepreneurial personality: entrepreneurial Awareness/Proactivity, Entrepreneurial Creativity, Opportunism /Motivation and Vision. While actual entrepreneurship measures mostly the third stage. In this regard, Entrepreneurial Awareness/Proactivity and Opportunism/ Motivation are determinants of the first stage of entrepreneurial development (Discovery or opportunity identification), while Entrepreneurial Creativity and Vision are determinants of the second stage of entrepreneurial development (Opportunity (problem-solution) validation and development).

The high reliability of the scale was also supported by a factor analysis which revealed four factors in our sample, but only one of them was large (associates with most of the items) and the rest were rather small. These results, as well as the very high Cronbach’s α of the entire questionnaire (which was 0.90) may indicate that a higher order factor exists.

Total entrepreneurial potential tendency score was calculated by adding scores of all individual items. I have divided the participants to two groups. In order to allow comparison with the binary data for actual entrepreneurship as well as to intensify the effects, participants that scored above the total sample average score were classified as high entrepreneurial score and participants who scored below the total sample average score were classified as low entrepreneurial score. However, we also examined entrepreneurial tendency as continues variable.

#### 3.2.2 Big five personality scale (BFI) [61]

This scale consists of 44 items. Eight items assess Extraversion (E; e.g., "I see myself as someone who is talkative"); nine items assess Agreeableness (A; e.g., "I see myself as someone who tends to find fault with others"); nine items assess Conscientiousness (C; e.g., "I see myself as someone who does a thorough job"); eight items assess Neuroticism (N; e.g., "I see myself as someone who is depressed, blue"); and ten items assess Openness to Experience (O; e.g., "I see myself as someone who is original, comes up with new ideas"). Respondents were instructed to rate each statement on a 5-point Likert-type that ranged from completely disagree (1) to completely agree (5). Cronbach’s α’s were 0.77 for Extraversion, 0.74 for Agreeableness, 0.77 for Conscientiousness, 0.76 for Neuroticism, and 0.77 for Openness to Experience.

#### 3.2.3 Socio economic and demographic questionnaire

Participants were asked to provide background information on variables that are known to influence entrepreneurial tendency. Ancestors’ countries of birth were included. Using the entrepreneurship levels of the birthplace countries of the father or the mother of each participant (data obtained from [26]), as a proxy for the cultural legacies of both household couple regarding entrepreneurship, will uniquely allow us to examine the effect of parents’ culture on the tendency to become an entrepreneur. Income and being an Arab minority or being an immigrant were included since these factors may necessitate individuals to become entrepreneurs facing discriminatory hiring practices [62, 63]. Religion and degree of religiosity were also included as additional controls. Education was included as indication of human capital that may assist in the accumulation of knowledge, leading to the development of skills useful to entrepreneurs [64]. In Israel, like in many western countries, women acquire more education than men regardless of whether they will become entrepreneurs.

### 3.3 Statistical analyses

The univariate logistic analysis included a t-test for continuous variables and a chi-squared test for categorical variables. Statistically significant variables were included in a multivariate model (after ruling out potential multicollinearity). The odds ratios (OR) and the 95% confidence intervals (95%CIs) were derived from these models, controlling for confounding variables. Statistical analyses were conducted using SPSS. All data descriptions, sources and statistics are given in Table 1.

## 4. Results

### 4.1 Study 1

#### 4.1.1 Results

In this study, we used two comparable specifications, which shared a full set of social, economic, cultural and personality factors in a fully controlled specification. I used a reverse stepwise procedure in which I first entered gender as a single variant specification, then the entire set of controls (each and every one is based on the entrepreneurship literature as factors that were found to associate with entrepreneurship) and then removed the insignificant variables, one by one from the least significant. In the final fully controlled specifications remained variables that were significant at least in one specification.

The specifications differed in their dependent variable–actual entrepreneurs versus high entrepreneurial tendency. Both specifications had a relatively high explanatory power. Moreover, the estimations of gender were stable and robust to the numerous controls.

In addition, I examined the associations of the first stage of entrepreneurial development, using the scores for Entrepreneurial Awareness/Proactivity and Opportunism/ Motivation (determinants of the first stage of entrepreneurial development, Discovery or opportunity identification) with the second stage of entrepreneurial development, using the scores for Entrepreneurial Creativity and Vision (determinants of the second stage of entrepreneurial development, Opportunity or problem-solution validation and development). The results indicate a positive significant association (coefficient reached 0.75) while the first stage accounted for 44.8% of the variance in the second stage. Furthermore, the associations of gender with both the first stage of entrepreneurial development and the second stage of entrepreneurial development were significantly negative (coefficients were -1.973 and -2.515 respectively).

In support of H1, gender was negatively significant in both specifications expressing a significant entrepreneurship gender gap in all three stages of entrepreneurial development (see Model A-3, Table 2 and Model A-9, Table 4). Moreover, the entrepreneurship gender gap was not significantly reduced when comparing actual entrepreneurship versus the tendency to become an entrepreneur (the confidence intervals were partially congruent). As a robustness test, I used a sub sample of 646 participants with no family connection to each other and found these findings robust. In order to examine the stability of the results further we also examined an OLS specification using the entrepreneurial tendency as continues variable, without dichotomized the data into high or low entrepreneurial tendency. The results were stable stressing the significance of the entrepreneurial gender gap (see Model A-5, Table 2).

**Table 2:** Factors associated with entrepreneurial tendency.

Variable	Model A-1	Model A-2	Model A-3	Model A-4	Model A-5
	OR (95%CI)	OR (95%CI)	OR (95%CI)	OR (95%CI)	B (t)
**Number of observations**	1288	1288	1288	1288	1288
**Constant**	1.248+++	0.005+++	0.004+++	0.013+++	150.166+++
					(24.914)
**Gender**	0.586+++	0.434+++	0.432+++	0.051+++	-7.816+++
	(0.470–0.732)	(0.324–0.580)	(0.323–0.577)	(0.008–0.344)	(-6.962)
**Extraversion**	-	1.134+++	1.133+++	1.134+++	1.152+++
		(1.100–1.170)	(1.099–1.168)	(1.100–1.169)	(10.308)
**Agreeableness**	-	0.948+++	0.946+++	0.947+++	-0.515+++
		(0.918–0.979)	(0.918–0.976)	(0.919–0.976)	(-4.544)
**Neuroticism**	-	0.962+++	0.964+++	0.965+++	-0.594+++
		(0.935–0.991)	(0.938–0.991)	(0.939–0.992)	(-5.657)
**Openness to Experience**	-	1.188+++	1.187+++	1.150+++	2.006+++
		(1.156–1.221)	(1.156–1.220)	(1.107–1.194)	(22.509)
**Conscientiousness**	-	0.993	-	-	-
		(0.962–1.025)			
**Age**	-	0.977+++	0.977+++	0.977+++	-0.271+++
		(0.964–0.991)	(0.964–0.990)	(0.964–0.990)	(-5.074)
**Religious**	-	1.316+	1.316+	1.323++	1.570
		(0.998–1.736)	(0.998–1.735)	(1.003–1.745)	(1.450)
**Having Children**	-	0.727	0.725	0.701	-1.791
		(0.467–1.132)	(0.465–1.130)	(0.449–1.095)	(-1.017)
**Employee**	-	0.633++	0.629++	0.622+++	-4.789+++
		(0.442–0.907)	(0.440–0.900)	(0.435–0.889)	(-3.495)
**Unemployed**	-	0.465++	0.461++	0.466++	-6.602+++
		(0.250–0.866)	(0.248–0.857)	(0.250–0.869)	(-2.703)
**Entrepreneurial Course**	-	1.859+++	1.879+++	1.921+++	5.513+++
		(1.190–2.905)	(1.204–2.934)	(1.226–3.010)	(3.189)
**Father Entrepreneur**	-	1.478++	1.520+++	1.504+++	4.764+++
		(1.068–2.047)	(1.113–2.076)	(1.100–2.055)	(3.862)
**Mother Entrepreneur**	-	1.160	-	-	-
		(0.710–1.895)			
**Interaction of Openness to Experience and Gender**	-	-	-	1.061++	-
				(1.007–1.118)	
**Cox & Snell R Square**	0.017	0.333	0.333	0.335	0.507*
**Nagelkerke R Square**	0.023	0.444	0.444	0.447	0.503**

^+^ Significant at the 10 percent level.

^++^ Significant at the 5 percent level.

^+++^ Significant at the 1 percent level.

*R Square.

**Adjusted R Square.

Indeed, the non-restricted single variant specifications presented a preliminary reduced gender gap. The OR estimation for the gender variable was 0.586 in the entrepreneurial tendency specification (see Model A-1, Table 2) versus OR of 0.364 in the actual entrepreneurship specification (see Model A-6, Table 4). However, the estimations went in other directions at the fully controlled specification. The OR estimation for the gender variable insignificantly decreased to 0.432 in the entrepreneurial tendency specification (see Model A-3, Table 2) versus an insignificant increased OR of 0.415 in the actual entrepreneurship specification (see Model A-9, Table 4).

In summary, the results of the fully controlled specifications indicated that being a woman corresponds to a decrease in the prevalence of entrepreneurial tendency by 56.8 percent and to a decrease in the prevalence of entrepreneurship by 58.5 percent.

In additional support of H1, examining each of the four aspects of entrepreneurial tendency reveals that men have significantly higher scores (p<0.01) in three aspects: Entrepreneurial Awareness/Proactivity, Entrepreneurial Creativity and Opportunism/ Motivation. The scores for Vision were practically the same (see Table 3).

**Table 3:** Average and standard deviation of entrepreneurship and of entrepreneurship tendency scores and its four aspects, by gender.

Gender	Number of Participants	Number of Entrepreneurs	Average Entrepreneurship Tendency Score	SD Entrepreneurship Tendency Score	Average Entrepreneurial Awareness/Proactivity Score	SD Entrepreneurial Awareness/Proactivity Score	Average Entrepreneurial Creativity Score	SD Entrepreneurial Creativity Score	Average Entrepreneurial Opportunism/ Motivation Score	SD Entrepreneurial Opportunism/ Motivation Score	Average Entrepreneurial Vision Score	SD Entrepreneurial Vision Score
Men	570	166	210	28	28	6	57	10	61	10	64	7
Women	718	93	202	26	26	6	55	10	58	9	64	7
Total	1288	259	206	27	26	6	56	10	59	9	64	7
Difference	-	-	7.9+++	2	2.1+++	0.4	2.6+++	-0.2	3.1+++	0.7	0	0

Several social and economic controls were also important determinants in both specifications in the fully controlled models (see Models A-3, Table 2 and A-9, Table 4). Age was negatively significant predictor in both specifications, however based on subsampling according to age percentiles, above 50 years it becomes insignificant. Being a salary employee and being unemployed were negatively significant in both specifications. The variable for being a housekeeper was negatively significant in the actual entrepreneurship specification and insignificant in the entrepreneurial tendency specification. Wage level was positively significant in the actual entrepreneurship specification and insignificant in the entrepreneurial tendency specification models (see Model A-9, Table 4). Education, family status, having children (or number of children), being religious, belong to the non-Jewish minority and born outside of Israel, were insignificant in both specifications. Having a course in entrepreneurship (and therefore may affect the way the participant is thinking about entrepreneurship) was positively significant in both specifications. However, interaction analysis of these social and economic variables did not support a moderation effect on the gender association with entrepreneurship tendency or with actual entrepreneurship, disconfirming H2 and H3.

**Table 4:** Factors associated with actual entrepreneurship.

Variable	Model A-6	Model A-7	Model A-8	Model A-9
	OR (95%CI)	OR (95%CI)	OR (95%CI)	OR (95%CI)
**Number of observations**	1288	1288	1288	1288
**Constant**	0.410+++	1.319	1.675	1.729
**Gender**	0.364+++	0.484+++	0.484+++	0.415+++
	(0.274–0.483)	(0.335–0.701)	(0.337–0.697)	(0.282–0.609)
**Extraversion**	-	1.043++	1.044++	1.047++
		(1.005–1.082)	(1.008–1.082)	(1.010–1.085)
**Agreeableness**	-	1.008	-	-
		(0.967–1.051)		
**Neuroticism**	-	0.974	0.973	0.971
		(0.939–1.012)	(0.939–1.008)	(0.937–1.007)
**Openness to Experience**	-	1.025	1.025	1.025
		(0.995–1.055)	(0.995–1.055)	(0.995–1.056)
**Conscientiousness**	-	0.966+	0.968+	0.966+
		(0.929–1.005)	(0.933–1.005)	(0.930–1.003)
**Age**	-	0.981++	0.981++	0.981++
		(0.965–0.998)	(0.965–0.997)	(0.966–0.998)
**Religious**	-	0.740	0.739+	0.746
		(0.516–1.061)	(0.516–1.058)	(0.521–1.070)
**Having Children**	-	1.583	1.588	1.562
		(0.889–2.818)	(0.893–2.826)	(0.876–2.784)
**Employee**	-	0.043+++	0.043+++	0.043+++
		(0.028–0.066)	(0.028–0.065)	(0.028–0.66)
**Unemployed**	-	0.062+++	0.060+++	0.060+++
		(0.027–0.140)	(0.026–0.137)	(0.026–0.136)
**Home Maker**	-	0.078+++	0.076+++	0.079+++
		(0.027–0.220)	(0.027–0.216)	(0.028–0.226)
**Wage Level**	-	1.110+++	1.109+++	1.115+++
		(1.033–1.192)	(1.033–1.190)	(1.038–1.198)
**Entrepreneurial Course**	-	2.841+++	2.909+++	2.865+++
		(1.736–4.648)	(1.782–4.749)	(1.752–4.684)
**Father Entrepreneur**	-	1.792+++	1.965+++	1.775
		(1.213–2.647)	(1.353–2.854)	(1.212–2.601)
**Mother Entrepreneur**	-	1.642+	-	-
		(0.953–2.828)		
**Interaction of Mother Entrepreneur and Gender**	-	-	-	3.270+++
				(1.557–6.867)
**Cox & Snell R Square**	0.039	0.289	0.287	0.292
**Nagelkerke R Square**	0.062	0.457	0.454	0.462

^+^ Significant at the 10 percent level.

^++^ Significant at the 5 percent level.

^+++^ Significant at the 1 percent level.

Regarding the parental controls, against H4, the cultural controls were insignificant in both specifications in the fully controlled models (see Models A-3, Table 2 and A-9, Table 4).

In support of H5a (see Models A-3, Table 2 and A-9, Table 4), Extraversion was positively significant predictor in both specifications. Openness to Experience was positively significant predictor of entrepreneurial tendency while insignificant predictor of actual entrepreneurship (although become significant when replacing the variable with a dichotomy dummy variable based on the average or the median score for this trait). Neuroticism was negatively significant predictor of entrepreneurial tendency while insignificant predictor of actual entrepreneurship. Similarly, agreeableness was negatively significant predictor of entrepreneurial tendency but insignificant predictor of actual entrepreneurship. As opposed to H5a, Conscientiousness was negatively insignificant predictor in both specifications.

Against H5b, women’s Openness to experience reduces the entrepreneurial gender gap. Openness to experience moderated the effect of gender. However, the interaction of Extraversion (or any other trait) with gender was insignificant (see Model A-4, Table 2).

In support of H6, having a father entrepreneur (the role model effect) was positively significant in both specifications. In addition, having a father entrepreneur is associate with increase odds in the actual entrepreneurship specification by a larger magnitude (97%) than in entrepreneurial tendency specification (52%), although this odds ratio difference was insignificant (the confidence intervals were partially congruent). The interaction between having a father entrepreneur and gender was insignificant. Having a mother entrepreneur was insignificant in both specifications in the fully controlled models (see Models A-3, Table 2 and A-8, Table 4). However, the interaction between having a mother entrepreneur and gender was positively significant in the actual entrepreneurship specification (see Model A-4, Table 2). That is, having a mother entrepreneur increase the odds of women (but not of men) to become an entrepreneur by 227 percent.

#### 4.1.2 Discussion

Discrimination is evident where differences in treatments under equal conditions can be identified. However, it is difficult to conclude whether the discrimination process was by impeding the disadvantaged groups or by favoring the advantaged majority. Indeed, in a recent contribution to the literature, [65] found clear evidence for favoritism but not for discrimination by either nationality or gender. They randomly assigned graders to students’ examinations and found significant gender difference only for exams containing names. The effect was not in the women’s exams but rather a positive effect for the men’s exams. Therefore, difference in actual entrepreneurship, could have been wrongly interpreted as favoritism towards men due to more welcoming environment at the execution stage of entrepreneurship.

However, the results of study 1 suggest that (after controlling for personality traits, culture, education, income etc.) the entrepreneurship gender gap started at earlier stage of entrepreneurship development, namely at the discovery of opportunity identification and the opportunity validation stages.

The results suggest that women have low tendency towards entrepreneurship even before the execution stage. That is, the entrepreneurial gender gap is not only a question of more favorable environment towards men at the execution stage but have deeper and earlier factors which is prevailed in the entrepreneurial personality as measured through the entrepreneurial tendency.

Indeed, the results also suggest that men have significantly higher scores in Entrepreneurial Awareness/Proactivity, Entrepreneurial Creativity and Opportunism/ Motivation while the scores for Vision were similar. Since both Entrepreneurial Awareness/Proactivity and Opportunism/ Motivation are determinants of the first stage of entrepreneurial development, the results suggest that the first stage is indeed the most affected. Furthermore, the significant association of the first stage scores and the second stage scores indicate that the gender gap in the first stage is transmitted in part to the second stage. In the second study I will try to address what is the role of the parental personality channel versus the behavioral role model channel.

It seems that the second stage of entrepreneurial development, which is more related to Entrepreneurial Creativity and to Vision, is affected to a lesser extent (through Entrepreneurial Creativity but not through Vision). These results are therefore in line with [33] who did not find gender differences in innovation.

Furthermore, another finding supports the indication that the second stage of entrepreneurial development is less affected. That is, women’s Openness to Experience reduces the entrepreneurial gender gap. This finding is indirectly questioning another traditional argument that women are less inclined to take risks and therefore are less inclined to engage in entrepreneurship [2]. The results suggest that women are more open to experience, therefore perceive themselves as prone to take more risks, than men regarding entrepreneurship.

Since personality is significantly evolved at young age, the results direct the spot to the role of parents, which will be examined in study 2. Moreover, the focus on early age of the entrepreneurial development is supported by several prior studies. According to [42] an individual’s attachment orientation, significantly determined in early childhood, not only predicts entrepreneurship but also which type of entrepreneur he/she will become. In addition [26], found that culture, which starts to affect children from an early age, significantly affects countries’ entrepreneurship levels. Both studies therefore support the view that social forces are affecting entrepreneurs from early childhood (either from the inner social circle of parents or from outer social circle of culture) and are critical in determining children’s potential to become an entrepreneur and their entrepreneurial characteristics. Although, the insignificance of the cultural controls was surprising. However, 93.2% of the sample are of Jewish origin, which is characterized with a very high entrepreneurship level, that may blur the original cultural effect of the parents’ countries of birth.

Furthermore, differences in outcomes such as actual entrepreneurship can also be a result of differences in the behavior of women relative to men. That is, as additional factor which is not related to unequal terms for example when women internalize a view in which they will receive an unequal environment for their entrepreneurship although the actual environment is not necessarily unequal. Indeed [66], found that members of racial minority groups negotiated significantly lower salary increases than did members of the racial majority, but this effect was dramatically reduced when controlled for social ties to the organization. In line with Seidel et al., the results of study 1 suggest that women may have also adopted a perception of entrepreneurial disadvantage.

These results may echo the results of [32] who found that men reported higher opportunity evaluation than women when no gender stereotypical information was presented to them, whereas they both evaluated business opportunity equally favorable when the information contained gender neutral attributes [31]. However, the results of study 1 suggest that the gender differences are not restricted to difference in Entrepreneurial Awareness/Proactivity but emerge in Entrepreneurial Creativity and Opportunism/Motivation as well.

In addition, the results of study 1 addresses also the prime suspect of women’s discrimination–i.e., income, family and children. Wage level was indeed positively significant predictor of actual entrepreneurship. It is important to have resources that will help you move from the first or second stage of entrepreneurial development into the third stage of actual entrepreneurship. However, it seems that for women income does not support their third stage of actual entrepreneurship. Thus, underlining the advantage of men over women, given that men generally earn more and have more control over the family economic resources. In contrast, wage level was insignificant predictor of entrepreneurial tendency, thus not associate with decrease entrepreneurial tendency and suggesting that other factors contribute to the decrease’s entrepreneurial self-perception of women. The variables for education, family status, having children and the number of children were insignificant in both specifications, and did not interact with gender.

On the other hand, the results suggest an indirect negative effect of family dynamics. The negative associations of being a salary employee, being unemployed and being a housekeeper, may suggest that families who drive women to home or to a traditional salary employment may push down both their actual entrepreneurship and entrepreneurial tendency.

While older people are facing discriminatory practices and social exclusion [67–69], it seems that ageism does not significantly increase the entrepreneurial gender gap. In addition, since the study controlled for age than the possibility that the negative correlation between gender and the entrepreneurship tendency might reflect the effects of one’s birth cohort is very limited. That is, there is always the possibility that younger generations are more inclined toward entrepreneurship. However, I believe that this is not a major concern. Had there been a birth cohort effect, it should have been more pronounced in earlier born cohorts, leading to greater gender gap of the entrepreneurship tendency with age and therefore support an even greater gender gap associations than the associations I identified.

Regarding personality traits, the results for Openness to Experience, Neuroticism, Extraversion, and Agreeableness are in line with the empirical literature. However, the negative insignificant correlation between Conscientiousness and entrepreneurship tendency contrasts previous findings [40, 41]. Nevertheless, previous papers did not examine the correlation between the Big Five personality traits and the entrepreneurship tendency within the general population but compared entrepreneurs to managers. It is possible that the same negative correlation (although insignificant) that was found for Conscientiousness would have been found in managers as well, even though they differ from entrepreneurs on this trait.

Study 2 will shed more light on the sources of women’s perception as having a low tendency towards entrepreneurship by directing the spot to the role of parents as role models. As a starting point, I found a difference in parents’ role modeling in study 1. While having a father entrepreneur was positively significant in both specifications and having a mother entrepreneur was positively significant only to women, the parents’ cultural factor was insignificant in both models. These results emphasize the effect of parents as role models. Parent’s personality effect will be addressed in study 2.

The findings that entrepreneur fathers positively influence both entrepreneurial tendency and actual entrepreneurship, while entrepreneur mothers affect only daughters and only at the actual entrepreneurship stage, may raise concerns about the differential influence of fathers and mothers. Study 2 will try to reconcile this question.

### 4.2 Study 2

#### 4.2.1 Results

In this study, I also used two comparable specifications, which shared a full set of parentals, social, economic, cultural and personality factors in a fully controlled specification. The procedure was identical to the procedure in study 1 (reverse stepwise). The specifications differed in their dependent variable–actual entrepreneurs versus entrepreneurial tendency. Both specifications had a relatively high explanatory power. Moreover, the estimations were stable and robust to the numerous controls, minimizing the risk of omitted variables.

In support of H7, the entrepreneurial tendency of fathers and their child was positively and significantly associated (see Model B-5, Table 5). However, contrary to H7, there was no significant relations between entrepreneurial tendency of mothers and their child, although with positive tendency (see Model B-3, Table 5). As opposed to H7, both parents’ entrepreneurial tendency was uncorrelated to the actual entrepreneurship of their children.

**Table 5:** Factors associated with entrepreneurial tendency and parental associations.

Variable	Model B-1	Model B-2	Model B-3	Model B-4	Model B-5	Model B-6
	OR (95%CI)	OR (95%CI)	OR (95%CI)	OR (95%CI)	OR (95%CI)	OR (95%CI)
**Number of observations**	321	321	321	321	321	213*
**Constant**	0.005+++	0.000+++	0.001+++	0.000+++	0.000+++	0.000+++
**Gender**	0.392+++	0.375+++	0.394+++	0.382+++	0.383+++	-
	(0.210–0.734)	(0.199–0.705)	(0.211–0.736)	(0.201–0.726)	(0.201–0.733)	
**Extraversion**	1.075++	1.078++	1.078++	1.089+++	1.089+++	1.060
	(1.011–1.144)	(1.013–1.146)	(1.013–1.146)	(1.022–1.160)	(1.022–1.160)	(0.985–1.140)
**Agreeableness**	0.958	0.962	0.958	0.959	0.959	1.003
	(0.898–1.021)	(0.902–1.025)	(0.898–1.021)	(0.899–1.024)	(0.898–1.025)	(0.926–1.087)
**Neuroticism**	0.970	0.971	0.972	0.974	0.975	0.994
	(0.917–1.025)	(0.918–1.028)	(0.919–1.028)	(0.920–1.033)	(0.920–1.034)	(0.927–1.066)
**Openness to Experience**	1.266+++	1.262+++	1.264+++	1.256+++	1.261+++	1.308+++
	(1.189–1.349)	(1.184–1.345)	(1.186–1.347)	(1.179–1.337)	(1.182–1.344)	(1.201–1.424)
**Age**	0.943+	0.944+	0.941	0.949	0.952	0.983
	(0.883–1.008)	(0.882–1.010)	(0.880–1.007)	(0.886–1.016)	(0.889–1.020)	(0.900–1.072)
**Religious**	1.076	1.130	1.068	1.233	1.248	1.120
	(0.606–1.912)	(0.633–2.017)	(0.601–1.898)	(0.683–2.228)	(0.687–2.264)	(0.533–2.355)
**Having Children**	0.485	0.566	0.488	0.575	0.574	0.580
	(0.199–1.179)	(0.229–1.397)	(0.199–1.194)	(0.230–1.437)	(0.230–1.430)	(0.171–1.962)
**Employee**	0.348+	0.327+	0.311+	0.306+	0.333+	0.228+
	(0.100–1.208)	(0.092–1.159)	(0.088–1.093)	(0.089–1.052)	(0.096–1.157)	(0.049–1.059)
**Unemployed**	0.152++	0.146++	0.144+++	0.141+++	0.167++	0.150++
	(0.035–0.657)	(0.033–0.647)	(0.33–0.626)	(0.033–0.603)	(0.038–0.733)	(0.024–0.923)
**Entrepreneurial Course**	1.777	1.858	1.752	1.771	1.579	2.126
	(0.771–4.099)	(0.796–4.341)	(0.756–4.061)	(0.739–4.244)	(0.658–3.790)	(0.763–5.926)
**Father Entrepreneur**	1.261	-	-	-	-	-
	(0.690–2.303)					
**Mother Entrepreneur**	-	-	-	-	2.498++	-
					(1.119–5.574)	
**Father Entrepreneurial Tendency** **	-	1.012++	-	1.013++	1.012++	2.144++
		(1.001–1.023)		(1.002–1.024)	(1.001–1.024)	(1.009–4.554)
**Mother Entrepreneurial Tendency**	-	-	1.009	-	-	-
			(0.998–1.020)			
**Entrepreneurial Grandmother from Father side**	-	-	-	2.730+++	2.358++	-
				(1.311–5.685)	(1.111–5.001)	
**Cox & Snell R Square**	0.364	0.372	0.368	0.387	0.397	0.376
**Nagelkerke R Square**	0.486	0.497	0.491	0.517	0.530	0.509

*Women only.

**In Model B-6 Father Entrepreneurial Tendency used as a dichotomy variable and in all other Models as continues variable.

^+^ Significant at the 10 percent level.

^++^ Significant at the 5 percent level.

^+++^ Significant at the 1 percent level.

In support of H6, having an entrepreneurial grandmother from the father’s side of the family as well as having an entrepreneurial mother were positively associated with the child’s entrepreneurial tendency (see Model B-5, Table 5).

In sum, while gender accounted for 5.5% of the variance in actual entrepreneurship under a single variant model, it accounted to only 2.2% in the fully controlled model (see Model B-5, Table 5). Moreover, in this model the parental variables accounted for 4.5% of the variance in actual entrepreneurship.

Against H8 and H9, none of the interactions of gender and parents’ entrepreneurial tendency were significant. Furthermore, Against H9, testing a subsample of women only, the entrepreneurial tendency of fathers was positive significant predictor of their daughters’ entrepreneurial tendency, while the entrepreneurial tendency of mothers was not (see Model B-6, Table 5).

In addition, all the Big-5 traits of both parents were insignificant predictors.

#### 4.2.2 Discussion

The purpose of study 2 was to identify how parents contribute to their children entrepreneurial tendency. Study 1 disconfirmed the cultural channel influence, the results of study 2 tested the effects of the personality channel versus the behavioral role model channel. Indeed, the parental variables accounted for 4.5% of the variance in actual entrepreneurship, twice as much as gender which accounted for only 2.2%. Furthermore, the contribution of gender dropped from 5.5% of the variance in actual entrepreneurship under a single variant model, to only 2.2% in the fully controlled model.

The results suggest a limited effect of the personality channel. First, none of the parents’ Big-5 personality traits had a significant association with the children entrepreneurial tendency. Second, only the entrepreneurial tendency of fathers had a significant association with the children’ (including the daughters) entrepreneurial tendency. Third, neither the entrepreneurial tendency of fathers nor that of the mothers was significantly associated with the children actual entrepreneurship. That is, even the limited parental personality channel is transmitted only by fathers and only with the children’s entrepreneurial tendency.

In contrast, the results of study 2 reemphasize the behavioral role model channel.

Indeed, the results of both studies point to an advantages position of fathers’ role modeling. It seems that fathers are treated as more dominant possible entrepreneurial role model then mothers. Fathers may affect their sons and daughters both through their entrepreneurial tendency, and through the direct role modeling as actual entrepreneurs. The direct role modeling of fathers can also derive from the grandmother of their side of the family.

Furthermore, while supporting the contribution of having entrepreneurial mothers, the results suggest an additional role models in the family, i.e., grandmothers from the father’s side of the family. It is interesting that a possible role model of grandmothers from the mother’s side of the family was insignificant, stressing again the role modeling transmitted by fathers.

My interpretation is that the grandfathers’ position competes to some extent with the position of fathers. Therefore, when the fathers are entrepreneurs, they serve as the role models for their children, leaving no room for the entrepreneurial grandfathers. While, when the fathers are not entrepreneurs, they serve as the role models for their children as traditional salary employee, leaving again no room for the entrepreneurial grandfathers. In contrast, grandmothers from the fathers’ side of the family does not compete with the fathers’ position as possible role models and therefore can serve as additional source of role modeling. Regarding the insignificance of entrepreneurial grandmothers from the mother side of the family, I already noted that the results suggest that fathers are treated as more dominant possible entrepreneurial role model then mothers. Therefore, mothers cannot bring their mothers nor their fathers as additional source of role modeling.

In contrast, the results suggest that mothers have a more limited effect. Not only that their entrepreneurial personality has no significant association with their child personality, but also their direct role modeling is transmitted only to their daughters and only to women actual entrepreneurship but not to women entrepreneurial tendency. In addition, there were no alternative role model figures from the mothers’ side of the family.

The results also suggest that while fathers may affect all three stages of entrepreneurship, mothers may affect only the third, actual entrepreneurship stage.

The role modeling literature emphasizes the lack of possible women role models either in direct or in indirect environments, such as in textbooks or television [70, 71]. In this regard, lack of women role models can deter women from seeing themselves as taking men-dominated positions. However, the proximity of women role models may increase women participation in these fields.

This thesis goes beyond this line of thinking by examining whether role modeling of fathers has a differential association with their sons versus their daughters and whether role modeling of mothers has a differential association with their sons versus their daughters. The results of this thesis may be related to findings which indicated that the impact of role models on women’s participation is not related to increase in information but maybe to effect on women’s inspiration [70]. That is, a role model can serve as a source for knowhow imitation, for example how to perform as an entrepreneur, or as a source for inspiration for women to break the entrepreneurial gender gap.

In summary, are fathers responsible for the entrepreneurial gender gap? It is not claimed that they directly discriminate their daughters by any behavioral acts. Indeed, interaction analysis of the fathers’ role modeling with their children’s gender revealed no significant association. However, the results may suggest that fathers have very high impact on descendants’ entrepreneurship. First, only fathers appear to affect all three stages of the entrepreneurship development. Second, their role modeling is larger than that of the mothers.

In addition, the results suggest that mothers’ behavior is also accountable for the gender gap. First, their role modeling seems not to affect their sons. Second, other figures from their side of the family does not seem to serve as alternative role models. Third, their entrepreneurial personality does not seem to affect their children personality. Taken together, mothers are treated as possible entrepreneurial role models to a lesser extent than fathers. However, my data set does not allow to draw any conclusion whether this phenomenon is due to the behavior of mothers or due to norms and attitudes of the children.

So how do parents contribute to the entrepreneurial gender gap? I suggest that the dialogue of mother-entrepreneurs with their child instill and encourage the development of entrepreneurial personality. This dialogue may differ between sons and daughters. Maybe mothers do not include their entrepreneurship background in their dialog with their children and focus on behavior and norms, whereas fathers are discussing their entrepreneurship experience. Moreover, fathers may expose their children to alternative entrepreneurial role models (such as of other members of their family) while mothers are less inclined to do so. Since there are less mothers’ entrepreneurs than fathers’ entrepreneurs and more fathers with high entrepreneurial tendency than mothers, it is possible that mothers are less likely expose their children to alternative entrepreneurial role models. Therefore, mothers inherit the entrepreneurial gender-gap from one generation to the next.

Alternatively, maybe the family norms emphasize more the fathers’ economic contribution to the family on the expense of the mothers’ contribution. Therefore, affecting the family norms that men are supposedly more equitable as economic and business role models. The dissociation of income in the results (with positive significant association with actual entrepreneurship of men only) may hint to this line of thinking.

## 5. General discussion and concluding remarks

The research on gender differences in entrepreneurship have revealed many factors, which partially explain the differences. However, the literature has failed to explain the full extent of the entrepreneurial gender gap. Moreover, the current study shows in which of the three entrepreneurial stages the entrepreneurial gender gap emerges and how parents contribute to this gap. Indeed, the contribution of parents to the variance in actual entrepreneurship is twice as much as the contribution of gender while the latter is mitigated under the fully controlled specification including the parental role.

This research employed a novel empirical approach and used a very large data set in order to examine these two important questions. The findings suggest that the entrepreneurial gender gap is not solely an outcome of challenging environment at the execution level. The gender gap in actual entrepreneurship is a reflection of significant differences in entrepreneurial tendency, which is developed in the first and the second stages of the entrepreneurial trajectory. When women reach the third stage of entrepreneurial development, the execution stage, they have already acquired a self-perception of an incapable and incommensurate entrepreneurial personality. Evidently, women have lower self-perception of features of the first stage of entrepreneurial development (i.e., Awareness/Proactivity and Opportunism/ Motivation) and to a lesser degree it is so also in features relate to the second stage of entrepreneurial development (i.e., Creativity but not Vision).

The importance of examining self-perception variables was stressed by [2] who stated that "subjective perceptual variables have a crucial influence on the entrepreneurship propensity of women and account for much of the difference in entrepreneurial activity between the sexes" (p. 341). Continuing this line of thinking, this thesis suggests an important contributor for the difference in entrepreneurial self-perception between the sexes, namely the role modeling of the parents.

Since personality develops at young age, the prime contributor for the inferior entrepreneurial tendency may be the parents. However, as the theoretical model presented in Fig 1 suggests, parents are mediators of three channels that affect entrepreneurship. After controlling for the parental cultural channel and the parental entrepreneurial personality channel, the remaining results indicate a significant role modeling behavioral channel.

In this regard, the results suggest that both parents contribute to the self-perception of the hampered women entrepreneurial personality and that their contribution is affecting all four aspects of the entrepreneurial tendency.

Fathers appear to affect all three stages of the entrepreneurship development including actual entrepreneurship both through their role modeling as well as through their entrepreneurial personality. Mothers’ entrepreneurial personality on the other hand does not seem to affect their children entrepreneurial personality. Therefore, fathers’ role modeling is larger than that of mothers, and furthermore they even recruit to their help other entrepreneurial role models from their side in the family.

As the idiom goes, the proof of the pudding is in the eating. That is, assuming that fathers have larger effect than mothers on all three stages of the entrepreneurial development, we may infer that the disadvantageous of women in entrepreneurship may emerge from ineffective role modeling of father on daughters. The exact mechanism differentiates fathers’ transmission of entrepreneurship by role modeling for their daughters compared to their sons’ remains for a future research. A possible such research may record a live father-daughter’s dialogs regarding entrepreneurship. In the meantime, a note may be taken, according to the literature, explicitly or implicitly, parents emphasizing gender categories and the "appropriate" behavior to girls and boys [55]. It is possible that fathers are treated as more suitable entrepreneurial role models then mothers because of transmission of gender stereotypes by the parents. Such transmission emphasizes for example that fathers’ economic contribution to the family is more important than mothers’ contribution.

Mothers, seems to have a smaller effect as role models. In addition, it appears that mothers’ role modeling is affecting their daughters but not their sons. Why non-entrepreneurs with high entrepreneurial tendency fathers affect their children entrepreneurial personality while equivalent mothers do not remain also for a future research. Why role modeling of mothers does not seem to be significant enough to influence both sons and daughters while fathers do is another open question left for future research.

Two theories may add to the interpretation of the results. According to the Expectation States Theory (EST), when group members are not familiar with each other’s capabilities, they form a set of expectations regarding the skills of the other group members. The skills are evaluated according to the status characteristics of the group members. The more the members of a group are assessed as having a higher status, the more they are assessed as having the ability and skills to assist the group. Since the new members are not aware of the true abilities of the other members, they rely on external, prominent, and often nominal characteristics. Therefore, attributions that are irrelevant for assessment such as gender may affect the evaluation process.According to the Social Role Theory (SRT), society attributes different roles to men and women. Men are expected to hold agentic roles, which imply higher status. Women are expected to behave with communal characteristics. Therefore, whereas men are expected to be more entrepreneurial and proactive, women are expected to be more sympathetic and comforting. Both theories can stand behind the parents’ contribution to the self-perception of the hampered women entrepreneurial personality [72–74]. Indeed, some authors suggested that women perceive themselves as less self-capable than men [2].

These results are worrying indeed, and they are worse than results for elderly people. [24] show that as people age, they express reduced tendency to engage in entrepreneurial activity. However, this decline is quite limited, it weakens with age, and entrepreneurial activity is stabilized after age 50 especially for participants with above-median entrepreneurship tendency scores. In contrast, the result in the current study reveals a much stronger associations which are apparent for both below and above-median entrepreneurship tendency scores.

However, the optimistic interpretation of the current results may support the argument that the entrepreneurial gender gap is not a deterministic result of nature but rather the results of nurture. It is evident that men have a better entrepreneurial self-perception than women do (and therefore men are more likely to establish a new business) and that parental role modeling is an important predictor for these differences in entrepreneurial self-perception.

The results have also important policy implications, which leave ground for optimism. Policy makers who wish to promote women entrepreneurship as a possible channel to tackle discrimination in traditional labor markets need to realize that it might be too late to intervene at the third stage of entrepreneurial development. Governments should intervene much earlier.

For example, governments can facilitate mothers’ involvement in their daughters’ self-perception of women entrepreneurs. Furthermore, the education system as early as possible can recruit women entrepreneurs in order to present women role modeling, thereby increasing the importance of women entrepreneurs from the children point of view. The children toys industry in support of children education specialists can help to prepare mothers–daughters’ toys and plays that will help to improve mothers–daughters/sons’ dialogs, which is relevant to entrepreneurship.

In this regard, part of the results is encouraging. I found no significant gender difference in Vision, which is an important part of the entrepreneurial tendency. Moreover, the personality trait of Openness to Experience, which is crucial to entrepreneurship, is high among women and in fact reduces the entrepreneurial gender gap. Since Openness to Experience accounts for major part of the entrepreneurial tendency, it may allow an entrepreneurial expression under more welcoming environment for women entrepreneurs. Finally, the results stress the high importance of women role models. First, having a mother entrepreneur increase the odds of women (but not of men) to become an entrepreneur by high magnitude (227 percent). Second, having entrepreneurial grandmothers (from the father’s side of the family) contributes significantly to both sons and daughters’ entrepreneurship. Therefore, outside educational intervention has indeed tools to work on especially if the intervention focuses on presenting women role models.

Some caveats must be noted when interpreting the empirical results. First, I used a cross sectional design for an intergenerational study. [75] defines four central design criterions for a credible intergenerational study. Meeting these four criterions will allow to identify both the level of continuity in the behavior of interest as well as to explore mediating and moderation processes:

1. The use of prospective data of the first generation (G1) and the second generation (G2) involvement in the behavior of interest. However, Thornberry stress that if the behavior of interest is relatively static than the use of prospective designs may not be essential. Indeed, entrepreneurship tendency is relatively stable across the lifespan [76]. Furthermore, Thornberry also specifically stresses that using different reporters (that is self-report for G1, and another self-report for G2) minimizes methodological problems such as reliance on common source.
2. The measures of each generation should be independent and based on different reporters. Two aspects that were fully met in my study.
3. The use of comparable measures for G1 and G2 is another criterion met in my study.
4. The use of detailed prospective data on life course development and especially important is data on major life course events such as work, family formation etc. Indeed, I collected these major life course trajectories in a retrospective approach but there is no reason to assume that collecting these trajectories prospectively will have a different effect on entrepreneurship tendency than retrospective data collection. In addition, as Thornberry recommend, I controlled for age and used multiple birth cohorts of G2 and the results were stable and robust. Finally, Thornberry stress the importance of a large set of socio economic and demographic controls which was also included in my study.

Under this framework, I believe that the study design meet in essence Thornberry’s central design criterions. Therefore, in Thornberry’s words this study is in a strong position beyond a longitudinal study design to draw our conclusions.

Furthermore, I believe that there is no serious concern for reverse causality in any of the specification (except maybe for income, which its inclusion does not significantly affects the results) and this is especially true for the parental cultural, personality and behavioral channels.

Second, I used a self-report survey, which has its limits. However, the expected directions as identified in the entrepreneurship literature characterized the explanatory variables. As for the generalizability of the results, it is important to note that the sample consisted mostly of students in business programs in a private college. That is, participants that share many similarities from a socio demographic point of view. The general population is supposed to be more variant. Hence, our results in the current sample present the lower limit of estimates of the gender gap and the negative role of parents may be even stronger.

## Supporting information

S1 File (XLS)
